# Prognostic factors in first-line atezolizumab-bevacizumab treatment of intermediate or advanced hepatocellular carcinoma

**DOI:** 10.1371/journal.pone.0354176

**Published:** 2026-07-28

**Authors:** Kyowon Gu, Tae Wook Kang, Jaeseung Shin, Dong Hyun Sinn, Myung Ji Goh, Jung Yong Hong, Sangah Chi

**Affiliations:** 1 Department of Radiology and Center for Imaging Science, Samsung Medical Center, Sungkyunkwan University School of Medicine, Seoul, Republic of Korea; 2 Department of Medicine, Samsung Medical Center, Sungkyunkwan University School of Medicine, Seoul, Republic of Korea; 3 Statistics and Data Center, Research Institute for Future Medicine, Samsung Medical Center, Republic of Korea; Kaohsiung Medical University Hospital, TAIWAN

## Abstract

**Purpose:**

To analyze the prognostic factors influencing outcomes in hepatocellular carcinoma (HCC) patients initially treated with atezolizumab-bevacizumab (Ate-Bev).

**Methods:**

We conducted a retrospective study on 47 treatment-naïve HCC patients (40 men; median age, 56 years) who were treated with Ate-Bev between September 2020 and July 2022 and underwent at least one response assessment based on the Response Evaluation Criteria in Solid Tumors 1.1. We evaluated the clinical variables and imaging features of pre-treatment magnetic resonance imaging (MRI). Using multivariable Cox hazard regression analysis, we analyzed prognostic factors for progression-free survival (PFS).

**Results:**

During the follow-up period (range, 1.5–24.9 months), 33 patients (70.2%) experienced tumor progression. The median PFS was 4.3 months (95% confidence interval [CI], 2.8–8.5 months). Nine patients received concomitant radiotherapy. Multivariable analysis indicated that younger age (hazard ratio [HR], 0.95; 95% CI, 0.92–0.99; *P* = 0.013), absence of concomitant radiation therapy (HR, 0.22; 95% CI, 0.07–0.69; *P* = 0.009), tumor extent ≥10 cm (HR, 2.54; 95% CI, 1.17–5.55; *P* = 0.019), and peritumoral arterial-phase hyperenhancement on the MRI (HR, 2.15; 95% CI, 1.00–4.63; *P* = 0.049) were factors associated with poor PFS.

**Conclusions:**

Age, concomitant radiation therapy, tumor extent, and peritumoral arterial-phase hyperenhancement on MRI were associated with PFS in patients with HCC who were initially treated with Ate-Bev.

## Introduction

Hepatocellular carcinoma (HCC) is one of the most common malignancies and a leading cause of cancer-related mortality worldwide [1]. Although early-stage disease can be cured by local ablation, surgery, or liver transplantation, a considerable number of patients still present with advanced disease at the time of diagnosis and have a poor prognosis [2]. Sorafenib was introduced as the first targeted systemic therapy to show efficacy against advanced HCC in the SHARP trial [3].

Recently, various combinations of immune checkpoint inhibitors have emerged as effective systemic treatments for HCC [1]. Among them, the combination of atezolizumab, programmed cell death-ligand 1 antibody, and bevacizumab, the vascular endothelial growth factor A antibody, (atezolizumab and bevacizumab [Ate-Bev]) is recommended as first-line treatment for advanced HCC according to the international guidelines for HCC management [4,5] because it confers superior survival benefits compared to sorafenib (19.2 months vs. 13.4 months) according to the results of the IMbrave-150 trial [6].

Despite the promising therapeutic potential of this new treatment, the factors determining the response to Ate-Bev are still largely unclear, thus constituting a significant barrier to the advancement of HCC treatment strategies. In general, a high tumor burden, which can be visualized using imaging studies, can have a negative effect on anti-cancer immunity, which can lead to a poor treatment response to immune checkpoint inhibitors [7]; however, to the best of our knowledge, few data exist regarding the analysis of prognostic factors for Ate-Bev using pre-treatment imaging findings in treatment-naïve patients, not in those with recurrent HCC [8]. Analyzing treatment-naïve patients is more effective for evaluating the genuine treatment effect of Ate-Bev compared to analyzing recurrent HCC patients, as it eliminates the confounding effects of prior treatments.

We examined prognostic factors for progression-free survival (PFS) after Ate-Bev treatment using certain features visible on contrast-enhanced magnetic resonance imaging (MRI), as well as clinical findings in treatment-naïve patients with intermediate or advanced HCC.

## Material and Methods

This retrospective case-control study was performed at a tertiary academic cancer center, Samsung Medical Center, Seoul, Republic of Korea. It was approved by an Institutional Review Board (SMC 2023-04-098); informed consent was waived owing to the retrospective nature of the study, which used de-identified data from the electronic medical records system. The data were accessed from May 2, 2023 to May 1, 2024 for research purposes.

### Patients

**Fig 1:**
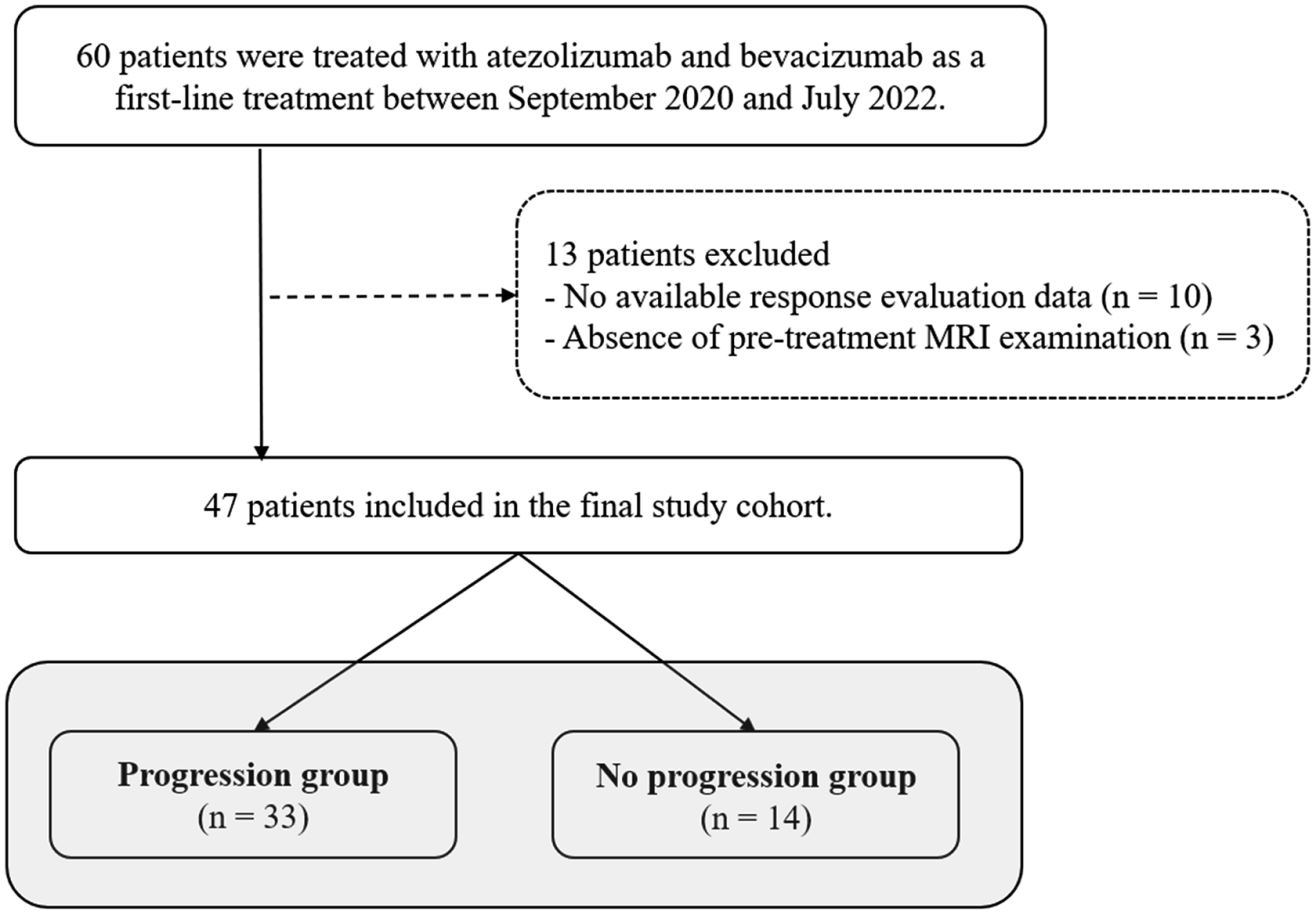
Patient selection process. MRI, Magnetic Resonance Imaging.

Between September 2020 and July 2022, 60 patients with intermediate or advanced HCC (Barcelona Clinic Liver Cancer stage [BCLC] B or C) underwent combination therapy with Ate-Bev as a first-line treatment at our institution [4]. The diagnostic criteria for HCC were based on the Korean Liver Cancer Study Group-National Cancer Center Korea practice guidelines for the management of HCC [9]. Treatment was administered to patients classified as Child-Pugh class A or B, with an Eastern Cooperative Oncology Group performance status score of 2 or less at the time of staging work-up. First-line treatment (treatment-naïve) was defined as systemic therapy administered to patients who had received no prior treatment for HCC, including locoregional or systemic therapy, before initiation of the study treatment, except for patients receiving concomitant radiation therapy for HCC within 2 months of initiating Ate-Bev treatment for combination therapy. In general, the decision to use concomitant radiation was made after discussion at a multidisciplinary tumor board and was based on the presence of significant vascular invasion that was considered potentially amenable to local control in conjunction with systemic therapy. Thirteen patients were excluded because of missing response evaluation data from the first treatment cycle (n = 10) and absence of MRI examination results within 2 months before treatment (n = 3); 47 patients (40 men; median age, 56 years) were included (Fig 1).

### MRI protocols

MRI was performed using a 3.0 Tesla MRI system (Achieva; Philips Healthcare, Best, The Netherlands). The baseline MRI protocol included the following sequences: a T1-weighted three-dimensional dual gradient-echo sequence, breath-hold multi-shot T2-weighted sequence, diffusion-weighted imaging, and a respiratory-triggered single-shot T2- and heavily T2-weighted sequence. For the dynamic enhancement study, images were acquired using a T1-weighted 3D gradient-echo sequence with spectral attenuated inversion recovery and fat suppression. The dynamic studies consisted of an unenhanced phase, arterial phase (20–35 s), portal phase (60 s), transitional phase (3 min), and hepatobiliary phase (HBP; 20 min). The optimal time for the arterial-phase imaging was determined using MR fluoroscopic bolus detection [10]. Gadoxetic acid as a contrast agent (Gd-EOB-DTPA, Primovist® [Eovist, United States], Bayer HealthCare, Berlin, Germany) was automatically administered intravenously at 1 mL/sec (0.025 mmol/kg body weight), followed by a 20-mL saline flush. MR parameter details are provided in **Supplementary table 1**.

### Treatment and assessment protocol

The patients were treated based on the standard dose of Ate-Bev (1200 mg atezolizumab plus 15 mg/kg per body weight bevacizumab intravenously, every 3 weeks) as used in the IMbrave-150 trial [6]. Before starting Ate-Bev treatment, patients who had not undergone esophagogastroduodenoscopy in the past 6 months underwent this procedure to screen for esophageal varices. If esophageal varices were identified, ligation was performed to reduce the risk of bleeding before treatment initiation. Beta-blocking agents were also used, and the dosage of bevacizumab was reduced from 15 mg/kg to 5–10 mg/kg during Ate-Bev treatment in patients who either had a high-risk varix or were on ribaroxaban for atrial fibrillation (6/47 patients; 12.8%). Ate-Bev treatment was continued unless the patient experienced intolerable toxicity or disease progression. The treatment response evaluation was routinely performed after two or three chemotherapy cycles.

### Image analysis

MRI images were independently reviewed by two board-certified radiologists (K. G. and T. W. K., with 7 and 15 years of experience in abdominal radiology, respectively) who were blinded to the clinical outcomes. After independent evaluation by both reviewers, the interobserver agreement was measured. Discordant imaging evaluations between the reviewers were resolved by consensus for multivariable analysis of prognostic factors. If a patient had multiple hepatic tumors, the largest index tumor was evaluated. The quality of the arterial-phase MRI was assessed based on a five-point scale by both reviewers due to using gadoxetic acid as an MR contrast agent (**Supplementary material**) [11]. All imaging analysis was performed using a picture archiving and communication system (Centricity; GE Healthcare).

The following MRI features were evaluated; *(a)* tumor extent, *(b)* tumor number (single or multiple), *(c)* the extent of portal vein tumor thrombosis, *(d)* hepatic vein tumor thrombosis, *(e)* bile duct invasion, *(f)* arterial-phase hyperenhancement (APHE), *(g)* portal-phase washout, *(h)* enhancing capsule, *(i)* tumor margin, *(j)* targetoid Liver Imaging Reporting & Data System-M features, *(k)* blood product in mass, *(l)* peritumoral APHE, *(m)* peritumoral HBP hypointensity, and *(n)* presence of bilobar involvement. Tumor extent was defined as the longest axial diameter measured across the entire liver tumor, encompassing small satellite nodules that are close to the index tumor on axial scans (< 2 cm) [12]. The extent of tumor thrombosis in the portal vein was classified as Vp0, Vp1, or Vp2 versus Vp3 or Vp4 (Vp0, no portal vein tumor thrombosis; Vp1, peripheral segmental portal vein; Vp2, second-order (segmental) portal vein branches; Vp3, first-order (lobar) portal vein branches; and Vp4, main trunk and/or contralateral portal vein branch to the primarily involved lobe) [13]. Bile duct invasion was considered positive if an enhancing tumor was directly demonstrated or if the tumor thrombus was visualized within the dilated duct. The following established prognostic factors were used for MRI-based aggressiveness assessments [14]; the tumor margin, determined as smooth when the entire margin was uninterrupted, free from irregularities or projections, and well-defined or non-smooth on HBP; peritumoral APHE, defined as a detectable portion of crescent- or polygonal-shaped enhancement outside the margin contacting the border of the tumor in the arterial phase, which becomes isointense with the background parenchyma in the delayed phase [15]; and peritumoral HBP hypointensity, defined as non-mass-like hypointensity of the liver adjacent to a mass in the HBP [16]. Other features were defined according to the computed tomography/MRI Liver Imaging Reporting & Data System version 2018 [17].

### Data collection and study outcomes

Before Ate-Bev treatment, the following data regarding baseline clinical and tumor characteristics were obtained using an electronic medical records system: *(a)* etiology of chronic liver disease, *(b)* BCLC stage, *(c)* Child-Pugh class, *(d)* modified albumin-bilirubin grade, *(e)* neutrophil-to-lymphocyte ratio, *(f)* presence of concomitant radiation therapy, *(g)* presence of extrahepatic metastasis, and *(h)* serum alpha-fetoprotein, and protein Induced by vitamin K absence or antagonist-II level.

Tumor responses were evaluated using the Response Evaluation Criteria in Solid Tumors, version 1.1 (RECIST 1.1) [18], and all treatment-related adverse events were classified according to the National Cancer Institute Common Terminology Criteria for Adverse Events version 5.0 [19]. PFS was defined as the time from initial Ate-Bev treatment to disease progression or death from any cause, whichever occurred first. The observation time for PFS was defined as the interval between the time of initial treatment and either the event or last visit to the outpatient clinic before March 15, 2023.

### Statistical analysis

Baseline clinical and imaging characteristics were compared using a two-sample *t*-test or Wilcoxon rank-sum test for continuous variables and the Chi-square test or Fisher’s exact test for categorical variables, according to their normality. PFS rates were estimated using the Kaplan-Meier method. Cox proportional hazard models were used to identify prognostic factors for PFS. The variables used for outcome analysis included sex, age, etiology of liver disease, BCLC stage, Child-Pugh class, neutrophil-to-lymphocyte ratio, concomitant radiation therapy, serum alpha-fetoprotein levels, tumor extent, extent of portal vein invasion, peritumoral APHE, and peritumoral HBP hypointensity. For tumor extent, the optimal cutoff of tumor extent for PFS was 9.8 cm (hazard ratio [HR], 2.18; 95% confidence interval [CI], 1.08–4.39; *P* = 0.029), which achieved the highest C-index and integrated area under the curve. We set the distance to 10 cm instead of 9.8 cm for practical use in a real clinical setting (**Supplementary material**). To evaluate the robustness of this threshold, tumor extent was additionally analyzed as a continuous variable in Cox regression models. Variables with *P* < 0.2 in the univariable analysis were considered potentially significant and subsequently incorporated in multivariable analyses. Given the limited number of progression events, the number of variables included in the final multivariable model was restricted to avoid overfitting. Firth’s penalized Cox regression models were used because of sparsity at some levels. To assess model performance and potential overfitting, internal validation of the final multivariable Cox model was performed using bootstrap resampling with 1,000 iterations. The optimism-corrected Harrell’s C-index was calculated to estimate the model’s discrimination ability. To evaluate the possibility that the observed age effect reflects underlying etiologic differences in an HBV-endemic population, we additionally included the etiology of liver disease in the multivariable model. For interobserver agreement analysis, the intraclass correlation coefficient for continuous variables and Cohen’s kappa for categorical variables were calculated for the selected imaging findings in the final multivariable analysis. Statistical significance was set at *P* < 0.05. All statistical analyses were performed using R Statistical Software (version 4.2.2; Foundation for Statistical Computing, Vienna, Austria).

## Results

### Patients and tumor characteristics

A total of 47 patients (40 men, 7 women; mean age 56.4 years; range 33–76 years) were included. The baseline patient characteristics are presented in **Table 1**. There was no discontinuation of Ate-Bev treatment due to serious adverse events during follow-up. Forty-two patients (89.4%) presented with multiple tumors, whereas only five (10.6%) had a single tumor. Portal vein tumor thrombosis was observed at stages Vp3 or Vp4 in 28 patients (59.6%), and extrahepatic metastasis was present in 29 patients (61.7%). 11 patients with BCLC stage B (23.4%) received systemic therapy because they were considered unsuitable for TACE or other locoregional therapies; seven exhibited infiltrative tumor growth, and four had high tumor multiplicity (> 10 nodules). Nine patients underwent concomitant radiation therapy for tumor thrombi in the portal vein (n = 8) and the hepatic vein (n = 1). In all nine cases, radiation therapy was directed at vascular tumor thrombosis, and three patients additionally received radiation to the index tumor. The radiation was delivered at a median dose of 30 Gy (range, 16–50 Gy) in a median of 10 fractions (range, 4–10). The non-progression group had a higher rate of concomitant radiation therapy with Ate-Bev treatment than the progression group (42.9% vs. 9.1%; *P* = 0.013). The other baseline clinical and tumor characteristics did not differ significantly between the two groups (*P* > 0.05).

**Table 1:** Baseline clinical and tumor characteristics at the time of initial atezolizumab-bevacizumab treatment.

	Total (n = 47)	Progression (n = 33)	No progression (n = 14)	*P-*value
**Clinical characteristics**				
Sex, male	40 (85.1)	28 (84.9)	12 (85.7)	> .99
Age (years)^*^	56 [50, 64]	56 [50, 64]	56 [51, 68]	0.625
Etiology for liver disease				0.060
Hepatitis B virus	31 (66.0)	24 (72.7)	7 (50)	
Hepatitis C virus or others	10 (21.3)	4 (12.1)	6 (42.9)	
Alcoholic	6 (12.7)	5 (15.2)	1 (7.1)	
BCLC stage				0.710
Intermediate	11 (23.4)	7 (21.2)	4 (28.6)	
Advanced	36 (76.6)	26 (78.8)	10 (71.4)	
Child-Pugh Class				0.148
A	42 (89.4)	31 (93.9)	11 (78.6)	
B	5 (10.6)	2 (6.1)	3 (21.4)	
Modified ALBI grade				0.189
1	21 (44.7)	15 (45.5)	6 (42.9)	
2a	10 (21.3)	9 (27.3)	1 (7.1)	
2b	16 (34.0)	9 (27.3)	7 (50)	
NLR^*^	3.5 [2.5, 5.0]	3.7 [2.5, 5.1]	2.7 [2.3, 4.5]	0.454
Concomitant radiation therapy	9 (19.2)	3 (9.1)	6 (42.9)	0.013
Extrahepatic metastasis	29 (61.7)	18 (54.6)	11 (78.6)	0.222
**Tumor characteristics**				
Serum α–FP level (ng/ml) ^*^	2371 [39.8, 65958]	4335 [68.4, 93889]	272.0 [11.5, 7711.5]	0.079
Serum PIVKA-II level (mAU/ml) ^*^	5082 [962, 25220]	5170 [1910, 28645]	4580.5 [275, 15999.5]	0.573
Tumor number				> .99
Single	5 (10.6)	4 (12.1)	1 (7.1)	
Multiple	42 (89.4)	29 (87.9)	13 (92.9)	
Vp3 or Vp4 tumor thrombosis	28 (59.6)	21 (63.6)	7 (50)	0.585
Hepatic vein tumor thrombosis	19 (40.4)	13 (39.4)	6 (42.9)	> .99
Bile duct invasion	10 (21.3)	8 (24.2)	2 (14.3)	0.700

*Data are median values with interquartile ranges in parentheses. Unless otherwise noted, data are numbers of patients with percentages in parentheses. Two-sample t-test or Wilcoxon rank-sum test for continuous variables and the Chi-square test or Fisher’s exact test for categorical variables, according to their normality.

α–FP, Alpha-Fetoprotein; ALBI, Albumin-bilirubin; BCLC, Barcelona Clinic Liver Cancer; NLR, Neutrophil-lymphocyte ratio; PIVKA-II, Protein Induced by Vitamin K Absence-II.

### MRI analysis

MRI analysis showed that the median tumor extent in the progression group was slightly greater than that in the non-progression group; however, no statistically significant difference was observed (11.9 vs. 7.6 cm; *P* = 0.071). There were no significant differences in any other MRI findings, including peritumoral APHE and peritumoral HBP hypointensity, between the two groups (**Table 2**). The image quality of the MR arterial phase, measured on a 5-point scale, had a score of 1 in 20 patients (42.5%), 2 in 19 patients (40.4%), and 3 in 8 patients (17.0%) according to reviewer 1. Reviewer 2 assigned a score of 1–23 patients (48.9%), 2–18 patients (38.3%), and 3–6 patients (12.8%). No patients received a score of 4 or 5 from either reviewer indicating severe or extensive artifact with non-interpretable images.

**Table 2:** MRI Analysis at the time of initial atezolizumab-bevacizumab treatment.

	Total (n = 47)	Progression (n = 33)	No progression (n = 14)	*P-*value
Tumor extent (cm)^*^	9.8 [7.3, 14.8]	11.9 [8.8, 16.4]	7.6 [5.4, 13.1]	0.071
≥ 10 cm	23 (48.9)	19 (57.6)	4 (28.6)	0.134
Arterial-phase hyperenhancement	41 (87.2)	29 (87.9)	12 (85.7)	> .99
Portal-phase washout	46 (97.9)	32 (97.0)	14 (100)	> .99
Enhancing capsule	25 (53.2)	17 (51.5)	8 (57.1)	0.973
Ill-defined margin	25 (53.2)	18 (54.6)	7 (50)	> .99
Targetoid LR-M feature	17 (36.2)	11 (33.3)	6 (42.9)	0.772
Blood product in mass	23 (48.9)	16 (48.5)	7 (50)	> .99
Peritumoral APHE	29 (61.7)	22 (66.7)	7 (50)	0.455
Peritumoral HBP hypointensity	31 (66.0)	22 (66.7)	9 (64.3)	> .99
Bilobar tumor involvement	28 (59.6)	21 (63.6)	7 (50)	0.585

*Data are median values with interquartile ranges in parentheses. Unless otherwise noted, data are numbers of patients with percentages in parentheses. Two-sample *t*-test or Wilcoxon rank-sum test for continuous variables and the Chi-square test or Fisher’s exact test for categorical variables, according to their normality. If a patient had multiple tumors, the largest index lesion on axial diameter was evaluated for image analysis.

APHE, Arterial-phase hyperenhancement; HBP, Hepatobiliary phase; LR-M, Liver Imaging Reporting & Data System-M.

### Treatment outcomes

**Fig 2:**
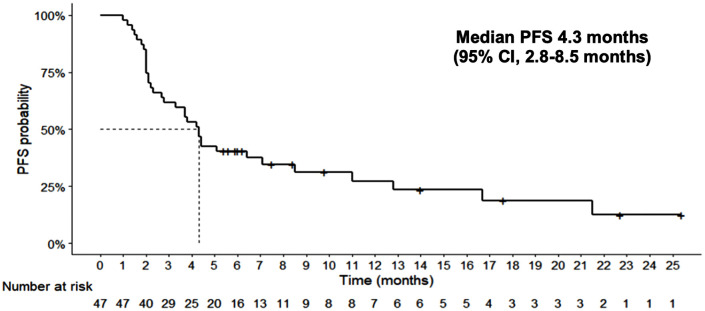
Progression-free survival of study patients (n  =  47). CI, confidence interval; PFS, progression-free survival.

As of March 15, 2023, there were a total of 33 patients (70.2%) who experienced tumor progression. Among them, 24 patients (72.7%) continued systemic treatment with different regimens, mostly sorafenib. The remaining nine patients were transitioned to best supportive care because of deteriorating liver function. Among the 33 patients with tumor progression, 13 (39.4%) experienced progression on their first response assessment, which was carried out after two or three cycles of therapy. The 1- and 2-year PFS rates were 27.3% (95% CI, 16.1%–46.2%) and 12.5% (95% CI, 4.2%–37.3%), respectively, in the study patients (n = 47). Median PFS was 4.3 months (95% CI, 2.8–8.5 months) (Fig 2). During follow-up, death occurred in four of the 33 patients (12.1%) in the progression group (median 4.4 months; range 1.7–12.4 months) and in two of the 14 patients (14.3%) in the non-progression group (median 8.5 months; range 4.2–12.8 months).

### Risk factor analysis for progression-free survival

**Fig 3:**
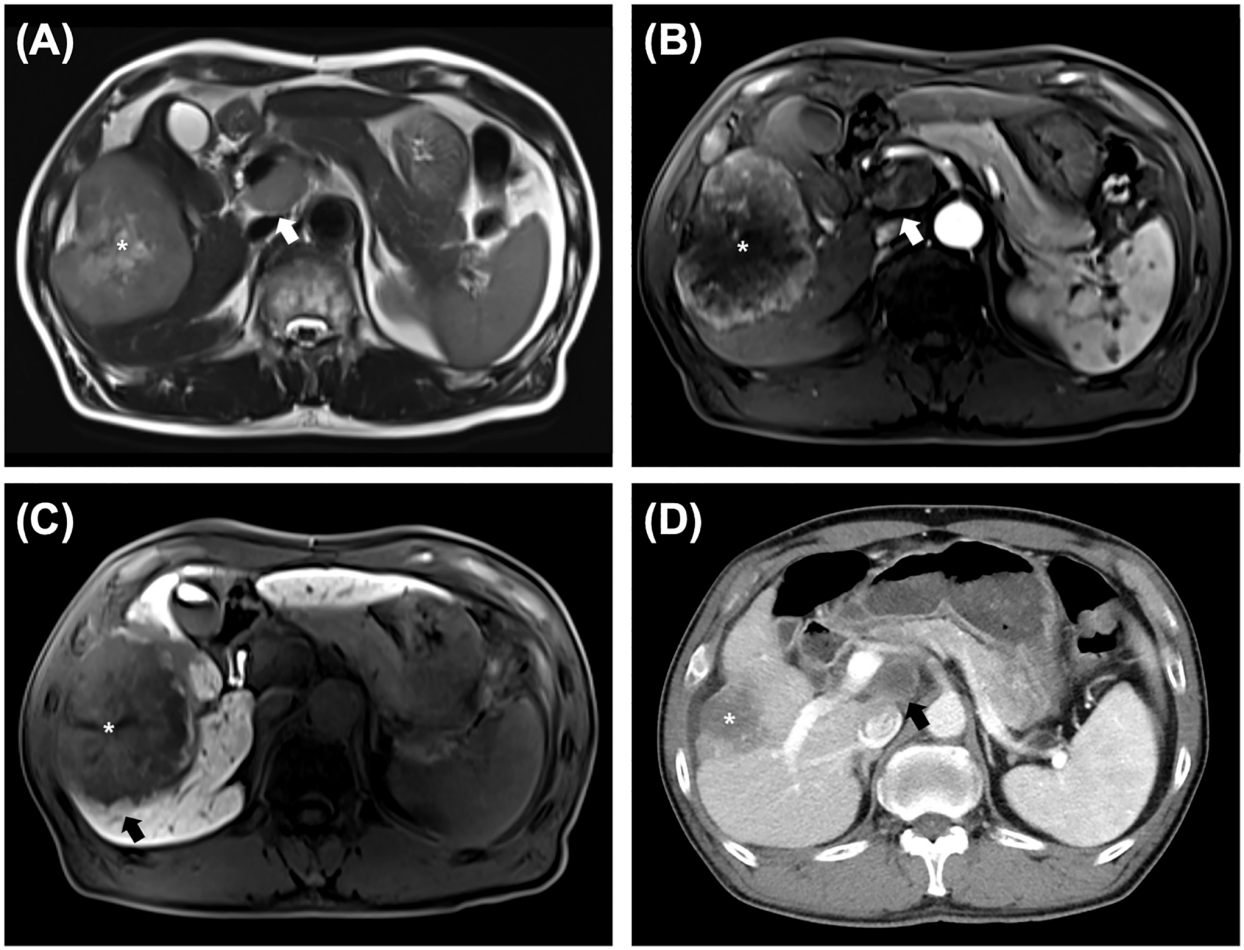
Images in a 62-year-old man with advanced hepatocellular carcinoma. (a) On T2-weighted magnetic resonance (MR) imaging, an 8.3 cm sized mass (asterisk) was seen in the right hepatic lobe. A metastatic lymph node is present in the portal space (white arrow). The mass was confirmed to be an Edmondson grade III hepatocellular carcinoma by biopsy. (b) The arterial-phase MR image shows an absence of peritumoral arterial-phase hyperenhancement around the index tumor (asterisk). Metastatic lymph nodes also show irregular enhancement (white arrow). Portal vein tumor thrombosis was not observed (not shown). (c) In the hepatobiliary phase, peritumoral hypointensity (black arrow) is observed around the index tumor (asterisk). (d) Computed tomography obtained during the portal phase 16.7 months after the atezolizumab-bevacizumab treatment without concomitant radiation therapy shows a shrunken HCC in the right hepatic lobe (asterisk) without other new metastatic lesions.

**Fig 4:**
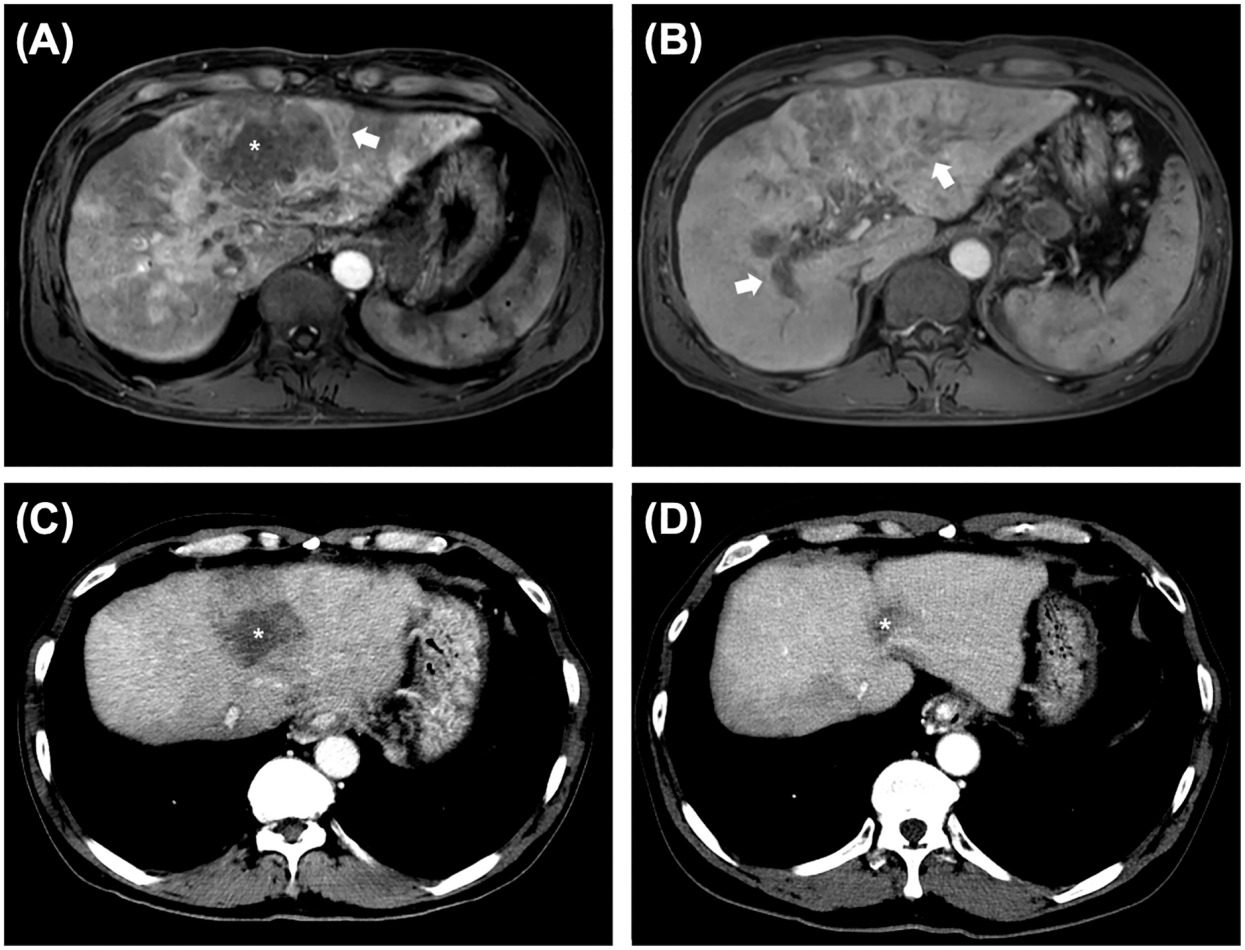
Images in a 50-year-old man with advanced hepatocellular carcinoma. (a) Arterial-phase MR image, a 9 cm sized index tumor (asterisk) with peritumoral arterial-phase hyperenhancement in liver segments III and IV. (b) Portal-phase MR reveals extensive portal vein tumor thrombosis in the left, right, and main portal veins, indicating Vp4 tumor thrombosis (white arrows). (c) Computed tomography (CT) obtained during the portal phase 3 months after atezolizumab-bevacizumab treatment without concomitant radiation therapy showed a shrunken HCC in the left hepatic lobe (asterisk) without new metastatic lesions. (d) CT obtained during the portal phase 21.5 months after the initial treatment shows an interval of more shrunken HCC in the left hepatic lobe (asterisk). The volume of extensive portal vein tumor thrombosis also decreased, and loss of its enhancement occurred with the development of cavernous transformation (not shown).

In the univariable analysis for PFS, age, concomitant radiation therapy, serum alpha-fetoprotein, tumor extent, and peritumoral APHE were incorporated into a multivariable analysis with *P* < 0.2. Among them, in the multivariable analysis, age (HR, 0.95; 95% CI, 0.92–0.99; *P* = 0.013), presence of concomitant radiation therapy (HR, 0.22; 95% CI, 0.07–0.69; *P* = 0.009), tumor extent ≥ 10 cm (HR, 2.54; 95% CI, 1.17–5.55; *P* = 0.019), and presence of peritumoral APHE (HR, 2.15; 95% CI, 1.00–4.63; *P* = 0.049) were significantly associated with PFS (**Table 3**) (Fig 3 **and Fig 4**). After bootstrap correction, the optimism-corrected C-index was 0.71 (95% CI, 0.62–0.80), indicating acceptable discrimination. When tumor extent was modeled as a continuous variable, it remained significantly associated with PFS (HR per 1-cm increase, 1.09; 95% CI, 1.01–1.17; *P* = 0.030), demonstrating consistency with the categorical cutoff-based analysis (**S1 Table**). After additional adjustment for etiology of liver disease, age remained significantly associated with PFS (adjusted HR, 0.95; 95% CI, 0.91–1.00; *P* = 0.034). The inter-reader agreement for tumor extent was moderately reliable (intraclass coefficient, 0.72; 95% CI, 0.55–0.83). In addition, agreement was almost perfect for binary tumor extent with a cutoff value of 10 cm (Cohen’s kappa, 0.87; 95% CI, 0.73–1.00) and substantial for peritumoral APHE (Cohen’s kappa; 0.72, 95% CI, 0.51–0.92).

**Table 3:** Multivariable risk factor analysis for progression-free survival.

	Progression-free Survival			
	Univariable		Multivariable	
Variable	HR (95% CI)	*P*-value	HR (95% CI)	*P*-value
Sex, male [female]	0.99 (0.38, 2.58)	0.984		
Age	0.98 (0.95, 1.01)	0.136	0.95 (0.92, 0.99)	0.013
Etiology of liver disease [HBV] Hepatitis C virus or others Alcoholic BCLC Stage, advanced [intermediate]	0.61 (0.27, 1.37) 1.95 (0.58, 6.55) 1.37 (0.62, 3.04)	0.215 0.231 0.280 0.440		
Child-Pugh Class B [A]	0.45 (0.11, 1.87)	0.270		
Neutrophil-Lymphocyte Ratio	1.11 (0.95, 1.30)	0.208		
Concomitant radiation therapy [no radiation therapy]	0.41 (0.14, 1.16)	0.092	0.22 (0.07, 0.69)	0.009
Serum α-FP (log)	1.07 (0.99, 1.17)	0.084	1.05 (0.96, 1.16)	0.309
Tumor Extent ≥ 10 cm [< 10 cm]	1.97 (0.99, 3.94)	0.054	2.54 (1.17, 5.55)	0.019
Vp3 or Vp4 Portal vein tumor thrombosis [none, Vp1, or Vp2]	1.43 (0.72, 2.86)	0.311		
Peritumoral APHE [Absence]	1.62 (0.80, 3.30)	0.182	2.15 (1.00, 4.63)	0.049
Peritumoral HBP hypointensity [Absence]	1.15 (0.57, 2.32)	0.698		

The reference category for each variable is shown in the square brackets.

All statistical analyses were conducted using the Cox proportional hazards model. Variables with *P* < 0.2 in the univariable analysis were considered potentially significant and subsequently incorporated in multivariable analyses.

α-FP, Alpha-fetoprotein; APHE, Arterial-phase hyperenhancement; BCLC, Barcelona Clinic Liver Cancer; CI, Confidence interval; HBP, Hepatobiliary phase; HBV, hepatitis B virus; HR, Hazard ratio

## Discussion

Our results showed that age, concomitant radiation therapy, tumor extent, and pre-treatment MRI findings were prognostic factors for tumor progression in patients with intermediate or advanced HCCs who had received Ate-Bev therapy as a first-line treatment.

Our median PFS (4.3 months) was shorter than that reported in the IMbrave150 trial [6]. This discrepancy likely reflects differences in baseline tumor burden and patient characteristics between our cohort and the IMbrave150 population. In our study, 76.6% of patients had BCLC stage C disease, and all patients were strictly treatment-naïve, with no prior locoregional therapy. Additionally, our cohort had a younger median age compared with the IMbrave150 population, and younger age was independently associated with shorter PFS in our analysis.

The extent of HCC, visualized using imaging studies, can reflect the tumor microenvironment. A large tumor burden can cause tissue hypoxia, creating an immunosuppressive microenvironment that may attenuate the effects of atezolizumab, an immune checkpoint inhibitor [20]. Additionally, the presence of a large tumor suggests that the host immune system is incapable of suppressing tumor growth, potentially making immune checkpoint inhibitors less effective [21]. In line with these assumptions, our results demonstrated that a tumor extent of 10 cm or larger indicated a poor response to Ate-Bev therapy. Similarly, a previous study [7] reported that larger tumor burden tends to diminish the effects of immune checkpoint inhibitors in various cancers.

The main limitation of imaging-based assessments of tumor burden, such as tumor size and number, is that the definition does not characterize the biological characteristics of each individual HCC because tumor grade and differentiation [22], molecular alterations [23], and the presence of microvascular invasion [24] can affect tumor aggressiveness and the patients’ clinical outcomes. The assessment of these factors requires tissue sampling through invasive biopsies or surgery; however, recent imaging techniques can provide insights into tumor biology in a non-invasive manner [14,16]. Among the imaging variables investigated in our study, peritumoral APHE was identified as an independent prognostic factor for PFS in the multivariable model. In general, peritumoral APHE in HCC can be seen as compensatory arterial hyperperfusion due to microscopic tumor thrombi of minute portal vein branches around the tumor, indicating microvascular invasion [24,25]. Given that in the present study, over 50% of the HCCs had Vp3 or Vp4 portal vein invasion and the mean tumor extent exceeded 10 cm, it is plausible that many patients already had microvascular invasion at the time of diagnosis. While these findings suggest a potential biological basis for the prognostic role of peritumoral APHE in patients receiving Ate-Bev treatment, the borderline statistical significance observed in the primary model (*P* = 0.049) and the loss of significance after additional adjustment for etiology in a sensitivity analysis (HR, 1.91; 95% CI, 0.84–4.38; *P* = 0.125; **Supplementary table**) suggest that this finding may not be robust to minor changes in model specification and therefore warrants validation in larger prospective cohorts.

In addition to imaging features, combined radiation therapy was associated with a lower risk of tumor progression in patients with HCC treated with Ate-Bev for HCCs. Although few studies examined this aspect [26,27], radiation therapy can create a synergistic effect against cancer cells in several different ways, especially in patients with portal vein tumor thrombosis [28], by driving a polyclonal T cell response, enhancing CD8^+^ T cell infiltration, provoking an interferon-γ response, and upregulating programmed cell death-ligand 1 [7,29]. However, the observed association should be interpreted with caution given the small number of patients who received radiation therapy in our cohort and the inherent selection bias associated with the retrospective study design, in which radiation therapy was selectively administered to patients with vascular tumor thrombosis. Therefore, these findings should not be interpreted as definitive evidence of a true synergistic therapeutic effect, and prospective studies with larger sample sizes are warranted to validate these observations. Regarding the other prognostic factors identified in our study, tumor progression tended to be less frequent among older patients. In contrast, in recent phase III trials of Ate-Bev treatment for HCC [30], an increasing population age of over 65 was associated with a lower objective response rate and reduced survival; however, owing to the variability in patient cohorts and differing outcomes (PFS vs. overall survival and objective response rate), further research is necessary for a more comprehensive interpretation.

Regarding the reliability of the imaging variables identified in our study, the method of measuring tumor extent (tumor extent ≥ 10 cm or not) was found to be more reliable than peritumoral APHE based on inter-reader variability (Cohen’s kappa; 0.87 vs. 0.72). This difference may be due to the subjective imaging interpretation of radiologists in cases such as peritumoral APHE [31], in contrast to the reliance on a simple binary measurement based on tumor extent. In addition, as the tumor size increases, imaging features become more heterogeneous [32], and various imaging findings appear complex. This could lead to lower inter-reader agreement for peritumoral APHE, given that the mean tumor extent in the current study exceeded 10 cm, as we included only patients with intermediate or advanced HCC undergoing systemic therapy. Thus, caution should be exercised when interpreting peritumoral APHE on MRI, considering substantial interobserver variability.

Beyond imaging and clinical variables, systemic inflammatory biomarkers have also been reported as prognostic indicators in patients receiving Ate-Bev therapy, particularly serum C-reactive protein (CRP)-based scoring systems. The CRAFITY, CABLE, and CRAPT-M scores have demonstrated significant associations with treatment outcomes in previous studies [33–35]. In our cohort, however, a substantial proportion of patients lacked available baseline serum CRP values (31/47, 65.9%) due to retrospective study. Given the role of systemic inflammation in the progression of HCC, the absence of comprehensive CRP data represents a limitation of our analysis. Future prospective studies should prioritize the systematic collection of inflammatory markers to better elucidate their synergistic impact on prognostication in patients undergoing Ate-Bev therapy.

This study had several limitations. First, given the retrospective nature of the cohort study, selection bias may have been present, including bias in the selection of radiation therapy. Second, Ate-Bev treatment has only recently been introduced as a first-line therapy for patients with advanced HCC (after previously being used in the second-line setting), making it challenging to include a sufficient number of patients for robust statistical analysis, particularly given the risk of overfitting in multivariable analysis. Third, although our primary outcome was PFS, the follow-up period was relatively short, preventing a comprehensive prognostic analysis of overall survival. However, considering that all patients have intermediate or advanced HCC and that there is a very high probability of progression in these patients (median PFS, 4.3 months), the current follow-up period is relatively sufficient for predicting PFS. Finally, our outcome measurements were based on the RECIST 1.1. Although modified RECIST has been introduced to overcome the limitation of RECIST 1.1 using the concept of “viable tumor” in the measurement of intrahepatic HCC lesions, a recent meta-analysis [36] showed that the performance of both RECIST 1.1 and modified RECIST in assessing disease progression, as our primary outcome, is not significantly different. Despite the abovementioned limitations, this is the first study to investigate imaging prognostic factors in patients receiving first-line Ate-Bev treatment for intermediate or advanced HCCs. While acknowledging the limitations of characterizing biological features without pathological specimens, our results suggest this approach as a non-invasive tool to aid clinicians in decision-making regarding treatment selection and patient prognostication in challenging clinical settings. Further research and validation studies are essential to refine and generalize our findings and enhance their clinical applicability.

In conclusion, younger age, absence of concomitant radiation therapy, larger tumor extent, particularly ≥ 10 cm, and the presence of peritumoral APHE on pre-treatment MRI were associated with a high risk of tumor progression in patients receiving Ate-Bev as a first-line treatment for intermediate or advanced HCCs.

## Supporting information

S1 File Assessment of motion artifact in arterial MR phase images. (DOCX)

S2 File Cut-off value analysis of tumor extent for progression-free survival. (DOCX)

S1 Table MR imaging sequences and parameters. (DOCX)

S2 Table Detailed measurement of all cut off value of tumor extent for progression-free survival. (DOCX)

S3 Table Sensitivity analysis for risk factor analysis. (DOCX)\

S3 File Anonymized study patient data. (XLSX)

## Abbreviations

APHE: arterial-phase hyperenhancement
Ate-Bev: atezolizumab and bevacizumab
BCLC: Barcelona Clinic Liver Cancer
CI: confidence interval
CRP: C-reactive protein
HBP: hepatobiliary phase
HCC: hepatocellular carcinoma
HR: hazard ratio
MRI: magnetic resonance imaging
PFS: progression-free survival
RECIST: Response Evaluation Criteria in Solid Tumors
