# Supplementary material for: Prognostic factors in first-line atezolizumab-bevacizumab treatment of intermediate or advanced hepatocellular carcinoma
Source: PLoS One. 2026 Jul 28;21(7):e0354176. doi: 10.1371/journal.pone.0354176 (PMC13412060; doi:10.1371/journal.pone.0354176)
Supplement: S1 File — (DOCX) [file pone.0354176.s001.docx]

***Assessment of motion artifact in arterial MR phase images.***

Both observers classified the error of motion artifacts in arterial magnetic resonance imaging using a five-point scale based on a previous study [1] as follows: score 1, none; score 2, minimal artifact with no effect on image interpretation; 3, moderate artifact with some images but no severe effect on image interpretation; score 4, severe artifact but images still interpretable; and score 5, extensive artifact and images non-diagnostic.

**References**

1. Davenport MS, Viglianti BL, Al-Hawary MM, Caoili EM, Kaza RK, Liu PSC, Maturen KE et al Comparison of acute transient dyspnea after intravenous administration of gadoxetate disodium and gadobenate dimeglumine: effect on arterial phase image quality. Radiology 2013; 266:452-461 https://doi: 10.1148/radiol.12120826 PMID: 23192781
