# Supplementary material for: Prognostic factors in first-line atezolizumab-bevacizumab treatment of intermediate or advanced hepatocellular carcinoma
Source: PLoS One. 2026 Jul 28;21(7):e0354176. doi: 10.1371/journal.pone.0354176 (PMC13412060; doi:10.1371/journal.pone.0354176)
Supplement: S2 File — (DOCX) [file pone.0354176.s002.docx]

***Cut-off value analysis of tumor extent for progression-free survival***

To subdivide patient’s tumor extent into two groups, we conducted a cox proportional hazards regression analysis and receiver operating characteristic analysis with C-index to obtain an optimal cutoff of tumor extent that can achieve the best progression-free survival. Based on the detailed measurement by all cut-points, the optimal cut-off of tumor extent for progression-free survival was 9.8 cm (hazard ratio, 2.18; 95% confidence interval, 1.08-4.39; *P*-value, 0.029), which achieved highest C-index and integrated area under the curve (**S2 Table**). We set a 10 cm instead of 9.8 cm for the sake of practical usability in a real clinical setting.
