# Supplementary material for: Prognostic factors in first-line atezolizumab-bevacizumab treatment of intermediate or advanced hepatocellular carcinoma
Source: PLoS One. 2026 Jul 28;21(7):e0354176. doi: 10.1371/journal.pone.0354176 (PMC13412060; doi:10.1371/journal.pone.0354176)
Supplement: S1 Table — (DOCX) [file pone.0354176.s003.docx]

**S1 Table.** MR imaging sequences and parameters.

| **Sequence** | **TR/TE (ms)** | **Flip angle (degree)** | **Section thickness (mm)** | **Matrix size** | **Bandwidth (Hx/pixel)** | **Field of view (cm)** | **Acquisition time (s)** |
| --- | --- | --- | --- | --- | --- | --- | --- |
| T1 weighted-2D dual GRE | 3.5/1.15-2.3 | 10 | 6 | 256 x 194 | 1918.6/0.226 | 32-38 | 14 |
| T1 weighted-3D GRE | 3.1/1.5 | 10 | 2 | 256 x 256 | 723.4/0.601 | 32-38 | 16.6 |
| Breath-hold multi-shot T2 weighted imaging | 1623/70 | 90 | 5 | 324 x 235 | 235.3/1.702 | 32-38 | 55 |
| Respiratory-triggered single-shot heavily T2 weighted imaging | 1156/160 | 90 | 5 | 376 x 270 | 388.9/1.117 | 32-38 | 120 |
| Diffusion-weighted images | 1600/70 | 90 | 5 | 112 x 112 | 79.5/5.467 | 32-38 | 126 |

2D (two-dimensional), 3D (three-dimensional), GRE (gradient echo), TR (repetition time), TE (echo time)
