# Supplementary material for: Prognostic factors in first-line atezolizumab-bevacizumab treatment of intermediate or advanced hepatocellular carcinoma
Source: PLoS One. 2026 Jul 28;21(7):e0354176. doi: 10.1371/journal.pone.0354176 (PMC13412060; doi:10.1371/journal.pone.0354176)
Supplement: S2 Table — (DOCX) [file pone.0354176.s004.docx]

**S2 Table**. Detailed measurement of all cut off value of tumor extent for progression-free survival.

| Cut-point  (cm) | Univariable Hazard Ratio | | | | C-index | | integrated AUC |
| --- | --- | --- | --- | --- | --- | --- | --- |
|  | Estimate | Lower | Upper | *P*-value | Estimate | Standard error |  |
| 3.8 | 0.220 | 0.028 | 1.720 | 0.1491 | 0.5148 | 0.0146 | 0.5234 |
| 4 | 0.203 | 0.044 | 0.938 | 0.0411 | 0.5295 | 0.0203 | 0.5365 |
| 4.5 | 0.664 | 0.157 | 2.813 | 0.5786 | 0.5148 | 0.0248 | 0.5222 |
| 4.6 | 1.743 | 0.412 | 7.371 | 0.4501 | 0.5037 | 0.0302 | 0.5324 |
| 4.7 | 1.176 | 0.355 | 3.900 | 0.7904 | 0.4858 | 0.0342 | 0.5176 |
| 5.3 | 1.585 | 0.482 | 5.214 | 0.4485 | 0.5016 | 0.0369 | 0.5345 |
| 5.7 | 1.345 | 0.473 | 3.830 | 0.5783 | 0.5005 | 0.0364 | 0.5284 |
| 6.7 | 1.676 | 0.590 | 4.765 | 0.3326 | 0.5163 | 0.0386 | 0.5452 |
| 6.8 | 1.530 | 0.592 | 3.956 | 0.3803 | 0.5205 | 0.0384 | 0.5429 |
| 6.9 | 1.416 | 0.586 | 3.421 | 0.4403 | 0.5232 | 0.0383 | 0.5394 |
| 7 | 1.929 | 0.791 | 4.705 | 0.1488 | 0.5416 | 0.0403 | 0.5696 |
| 7.6 | 2.183 | 0.896 | 5.316 | 0.0856 | 0.5564 | 0.0413 | 0.5830 |
| 7.7 | 1.925 | 0.868 | 4.271 | 0.1072 | 0.5680 | 0.0414 | 0.5789 |
| 8.2 | 1.760 | 0.819 | 3.782 | 0.1472 | 0.5643 | 0.0416 | 0.5722 |
| 8.8 | 1.965 | 0.914 | 4.223 | 0.0835 | 0.5790 | 0.0420 | 0.5852 |
| 8.9 | 1.852 | 0.883 | 3.885 | 0.1029 | 0.5796 | 0.0423 | 0.5805 |
| 9 | 1.657 | 0.806 | 3.406 | 0.1695 | 0.5648 | 0.0439 | 0.5683 |
| 9.1 | 1.634 | 0.806 | 3.313 | 0.1732 | 0.5574 | 0.0466 | 0.5719 |
| 9.2 | 1.821 | 0.897 | 3.696 | 0.0971 | 0.5722 | 0.0465 | 0.5847 |
| 9.3 | 1.950 | 0.969 | 3.925 | 0.0612 | 0.5875 | 0.0460 | 0.5900 |
| **9.8** | **2.179** | **1.082** | **4.389** | **0.0293** | **0.6022** | **0.0456** | **0.6028** |
| 10.6 | 1.974 | 0.989 | 3.937 | 0.0536 | 0.5875 | 0.0463 | 0.5909 |
| 10.8 | 1.758 | 0.887 | 3.485 | 0.1058 | 0.5653 | 0.0474 | 0.5779 |
| 11.9 | 1.636 | 0.830 | 3.227 | 0.1553 | 0.5569 | 0.0470 | 0.5697 |
| 12.1 | 1.520 | 0.772 | 2.992 | 0.2254 | 0.5474 | 0.0467 | 0.5611 |
| 12.3 | 1.459 | 0.740 | 2.876 | 0.2753 | 0.5453 | 0.0466 | 0.5560 |
| 12.5 | 1.369 | 0.692 | 2.707 | 0.3671 | 0.5379 | 0.0466 | 0.5485 |
| 14 | 1.428 | 0.719 | 2.837 | 0.3091 | 0.5506 | 0.0458 | 0.5495 |
| 14.4 | 1.363 | 0.680 | 2.732 | 0.3831 | 0.5469 | 0.0460 | 0.5432 |
| 14.5 | 2.181 | 1.079 | 4.405 | 0.0298 | 0.5822 | 0.0423 | 0.5886 |
| 14.6 | 1.956 | 0.959 | 3.991 | 0.0652 | 0.5632 | 0.0416 | 0.5770 |
| 15 | 1.984 | 0.958 | 4.111 | 0.0651 | 0.5659 | 0.0413 | 0.5743 |
| 16 | 2.512 | 1.210 | 5.216 | 0.0135 | 0.5822 | 0.0390 | 0.5905 |
| 16.4 | 2.339 | 1.109 | 4.936 | 0.0257 | 0.5711 | 0.0387 | 0.5809 |
| 17.2 | 2.125 | 0.985 | 4.586 | 0.0547 | 0.5564 | 0.0378 | 0.5683 |
| 17.4 | 2.161 | 0.934 | 4.997 | 0.0717 | 0.5532 | 0.0364 | 0.5580 |
| 17.5 | 1.803 | 0.742 | 4.382 | 0.1929 | 0.5300 | 0.0319 | 0.5448 |
| 20 | 2.117 | 0.810 | 5.531 | 0.1260 | 0.5390 | 0.0306 | 0.5451 |
| 20.7 | 1.553 | 0.470 | 5.128 | 0.4700 | 0.5126 | 0.0248 | 0.5255 |
| 22.3 | 1.046 | 0.249 | 4.405 | 0.9506 | 0.4916 | 0.0147 | 0.5124 |

AUC, the area under the receiver operating characteristic curve
