# Supplementary material for: Prognostic factors in first-line atezolizumab-bevacizumab treatment of intermediate or advanced hepatocellular carcinoma
Source: PLoS One. 2026 Jul 28;21(7):e0354176. doi: 10.1371/journal.pone.0354176 (PMC13412060; doi:10.1371/journal.pone.0354176)
Supplement: S3 Table — (DOCX)\ [file pone.0354176.s005.docx]

**S3 Table.** Sensitivity analysis for risk factor analysis

| Progression-free Survival | | |
| --- | --- | --- |
|  | Multivariable | |
| **Variable** | **HR (95% CI)** | ***P*-value** |
| Sex, male [female] |  |  |
| Age | 0.95 (0.91, 1.00) | 0.034 |
| Etiology for liver disease |  | 0.375 |
| HCC or others vs HBV | 0.80 (0.31, 2.05) | 0.640 |
| Alcoholic vs HBV | 2.59 (0.53, 12.69) | 0.240 |
| BCLC Stage, advanced [intermediate] |  |  |
| Child-Pugh Class B [A] |  |  |
| Neutrophil-Lymphocyte Ratio |  |  |
| Concomitant radiation therapy [no radiation therapy] | 0.17 (0.05, 0.59) | 0.005 |
| Serum α-FP (log) | 1.03 (0.92, 1.14) | 0.638 |
| Tumor Extent | 1.09 (1.01, 1.17) | 0.030 |
| Vp3 or Vp4 Portal vein thrombosis [none, Vp1, or Vp2] |  |  |
| Peritumoral APHE [Absence] | 1.91 (0.84, 4.38) | 0.125 |
| Peritumoral HBP hypointensity [Absence] |  |  |

The reference category for each variable is shown in the square brackets.

All statistical analyses were conducted using the Cox proportional hazards model. Etiology for liver disease as well as variables with *P* < 0.2 in the univariable analysis were included in multivariable analyses.

α-FP, Alpha-fetoprotein; APHE, Arterial-phase hyperenhancement; BCLC, Barcelona Clinic Liver Cancer; CI, Confidence interval; HBP, Hepatobiliary phase; HBV, Hepatitis B virus; HCC, Hepatitis C virus; HR, Hazard ratio
